# Oscillatory dynamics in a discrete predator-prey model with distributed delays

**DOI:** 10.1371/journal.pone.0208322

**Published:** 2018-12-26

**Authors:** Changjin Xu, Lilin Chen, Peiluan Li, Ying Guo

**Affiliations:** 1 Guizhou Key Laboratory of Economics System Simulation, Guizhou University of Finance and Economics, Guiyang 550004, PR China; School of Information Science and Engineering, Central South University, Changsha 410083, PR China; 2 School of Mathematics and Statistics, Guizhou University of Finance and Economics, Guiyang 550004, PR China; 3 School of Mathematics and Statistics, Henan University of Science and Technology, Luoyang 471023, PR China; 4 School of Information Science and Engineering, Central South University, Changsha 410083, PR China; North University of China, CHINA

## Abstract

This work aims to discuss a predator-prey system with distributed delay. Various conditions are presented to ensure the existence and global asymptotic stability of positive periodic solution of the involved model. The method is based on coincidence degree theory and the idea of Lyapunov function. At last, simulation results are presented to show the correctness of theoretical findings.

## Introduction

It is well known that the qualitative analysis of predator-prey models is an interesting mathematical problem and has received great attention from both theoretical and mathematical biologists [1–5]. In particular, the periodic solutions are of great interest. During the past decades, a great deal of excellent results have been reported for a lot of different continuous or impulsive predator-prey models. For example, Zhang and Hou [6] investigated the four positive periodic solutions of a ratio-dependent predator-prey system with multiple exploited (or harvesting) terms. Liu and Yan [7] considered positive periodic solutions for a neutral delay ratio-dependent predator-prey model with a Holling type II functional response. Liu [8] dealt with the impulsive periodic oscillation of a predator-prey model with Hassell-Varley-Holling functional response. For more related work, one can see [9–27]. Dunkel [28] pointed out that feedback control item in predator-prey models depends on the population number for certain time past and also depends on the average of the population number for a period of time past. In particular, time delay often occur in predator-prey models due to the impact of all the past life history of the predators and preys on their present birth rates. In many cases, the time delay will extend over the entire past due to the intra-species and inter-species competition. Then there is a distribution of delays over a period of time, thus the distributed delays should be incorporated in predator-prey models.

The functional response plays a key role in characterizing the interaction of predators and preys. Based on the experiments of different kinds of species, Holling [29] proposed three types of functional responses: (I) *f*_1_(*u*) = *au*, (II) $f_{2}\left(u\right)=\frac{au}{c+u},$ (III) $f_{3}\left(u\right)=\frac{au^{2}}{c+u^{2}},$ where *u*(*t*) represents the prey density at time *t*, *c* > 0 is the half-saturation constant, *a* > 0 denotes the search rate of the predator. Holling type II functional response is most typical of predators that specialized on one or a few prey [29–33]. So in this paper, Holling type II functional response is introduced in model (1).

Motivated by the viewpoint, we proposed the following predator-prey model with Holling II functional response and distributed delays

$$\begin{array}{c} \left\{ \begin{array}{c} \frac{dx_{1}}{dt}=x_{1}\left(t\right)[r_{1}\left(t\right)-\int_{-\infty }^{t}k_{1}\left(s-t\right)x_{1}\left(s\right)ds]\\ \;\;\;\;\;\;\;\;\;-\frac{x_{1}\left(t\right)}{1+mx_{1}\left(t\right)}\int_{-\infty }^{t}k_{2}\left(s-t\right)x_{2}\left(s\right)ds,\\ \frac{dx_{2}}{dt}=x_{2}\left(t\right)[-r_{2}\left(t\right)-\int_{-\infty }^{t}k_{3}\left(s-t\right)x_{2}\left(s\right)ds]\\ \;\;\;\;\;\;\;\;\;+x_{2}\left(t\right)\int_{-\infty }^{t}\frac{k_{4}\left(s-t\right)x_{1}\left(s\right)}{1+mx_{1}\left(t\right)}ds, \end{array}\right. \end{array}$$ (1)

where *x*_*i*_(*t*)(*i* = 1, 2) stands for the prey and predator density at time *t*, *r*_1_(*t*) denotes the intrinsic growth rate of prey at time *t* and *r*_2_(*t*) denotes the death rate of predator at time *t*, *m* > 0 stands for the half-saturation constant, *k*_*i*_: (−∞, 0] → (0, +∞)(*i* = 1, 2, 3, 4) is continuous function such that $\int_{-\infty }^{t}k_{i}\left(s\right)ds=1,\int_{0}^{\infty }sk_{i}\left(s\right)ds<\infty .$ For the biological meaning of model (1), one can see [34].

As pointed out in [35–42], discrete time models are more better to describe the dynamical behaviors than continuous ones since the populations have non-overlapping generations. What’s more, discrete-time systems can provide convenience for numerical simulations. Thus it is interesting to investigate discrete-time systems. The principle aim of this paper is to propose a discrete version of system (1) and analyze the effect of the periodicity of the ecological and environmental parameters on the dynamics of discrete time predator-prey model.

## Discrete version of system (1)

Following [40, 43] and assuming that the average growth rates in system (1) change at regular intervals of time, one has

$$\begin{array}{c} \left\{ \begin{array}{c} \frac{1}{x_{1}\left(t\right)}{\dot{x}}_{1}\left(t\right)=r_{1}\left(\left[t\right]\right)-\sum_{l=0}^{+\infty }k_{1}\left(-l\right)x_{1}\left(\left[t\right]-l\right)-\frac{1}{1+mx_{1}\left(\left[t\right]\right)}\sum_{l=0}^{+\infty }k_{2}\left(-l\right)x_{2}\left(\left[t\right]-l\right),\\ \frac{1}{x_{2}\left(t\right)}{\dot{x}}_{2}\left(t\right)=-r_{2}\left(\left[t\right]\right)-\sum_{l=0}^{+\infty }k_{3}\left(-l\right)x_{2}\left(\left[t\right]-l\right)+\sum_{l=0}^{+\infty }\frac{k_{4}\left(-l\right)x_{1}\left(\left[t\right]-l\right)}{1+mx_{1}\left(\left[t\right]\right)}, \end{array}\right. \end{array}$$ (2)

where [*t*] stands for the integer part of *t*, *t* ∈ (0, +∞) and *t* ≠ 0, 1, 2, ⋯. The solution $\bar{x}={\left(x_{1},x_{2}\right)}^{T}$ of (2) possesses the following natures:

1. $\bar{x}$ is continuous on [0, +∞).
2. $\frac{dx_{1}\left(t\right)}{dt},\frac{dx_{2}\left(t\right)}{dt}$ exist for ∀ *t* ∈ [0, +∞) with the possible exception of the points *t* ∈ {0, 1, 2, ⋯}, where left-sided derivative exists.
3. (2) holds ∀ [*k*, *k* + 1), where *k* = 0, 1, 2, ⋯.

Integrating (2) on [*k*, *k* + 1), *k* = 0, 1, 2, ⋯, one has

$$\begin{array}{c} \left\{ \begin{array}{c} x_{1}\left(t\right)=x_{1}\left(k\right)\exp\{[r_{1}\left(k\right)-\sum_{l=0}^{+\infty }k_{1}\left(-l\right)x_{1}\left(k-l\right)\\ \;\;\;\;\;\;\;\;\;\;\;-\frac{1}{1+mx_{1}\left(k\right)}\sum_{l=0}^{+\infty }k_{2}\left(-l\right)x_{2}\left(k-l\right)]\left(t-k\right)\},\\ x_{2}\left(t\right)=x_{2}\left(k\right)\exp\{[-r_{2}\left(k\right)-\sum_{l=0}^{+\infty }k_{3}\left(-l\right)x_{2}\left(k-l\right)\\ \;\;\;\;\;\;\;\;\;\;\;+\sum_{l=0}^{+\infty }\frac{k_{4}\left(-l\right)x_{1}\left(k-l\right)}{1+mx_{1}\left(k\right)}]\left(t-k\right)\}. \end{array}\right. \end{array}$$ (3)

Let *t* → *k* + 1, then (3) reads as

$$\begin{array}{c} \left\{ \begin{array}{c} x_{1}\left(k+1\right)=x_{1}\left(k\right)\exp\{[r_{1}\left(k\right)-\sum_{l=0}^{+\infty }k_{1}\left(-l\right)x_{1}\left(k-l\right)\\ \;\;\;\;\;\;\;\;\;\;\;\;\;\;\;\;-\frac{1}{1+mx_{1}\left(k\right)}\sum_{l=0}^{+\infty }k_{2}\left(-l\right)x_{2}\left(k-l\right)]\},\\ x_{2}\left(k+1\right)=x_{2}\left(k\right)\exp\{[-r_{2}\left(k\right)-\sum_{l=0}^{+\infty }k_{3}\left(-l\right)x_{2}\left(k-l\right)\\ \;\;\;\;\;\;\;\;\;\;\;\;\;\;\;\;+\sum_{l=0}^{+\infty }\frac{k_{4}\left(-l\right)x_{1}\left(k-l\right)}{1+mx_{1}\left(k\right)}]\}, \end{array}\right. \end{array}$$ (4)

which is a discrete version of system (1), where *k* = 0, 1, 2, ⋯.

The following assumptions are made:

(H1) *r*_*i*_: *Z* → *R*^+^ is positive *α*-periodic (*α* is a positive integer), i.e., *r*_*i*_(*k* + *α*) = *r*_*i*_(*k*)(*i* = 1, 2), ∀ *k* ∈ *Z*.

(H2) The following inequalities hold true.

$$\begin{array}{ccc} & & 0\leq \sum_{l=0}^{+\infty }k_{1}\left(-l\right)<+\infty ,0\leq \sum_{l=0}^{+\infty }k_{2}\left(-l\right)<+\infty ,\\ & & 0\leq \sum_{l=0}^{+\infty }k_{3}\left(-l\right)<+\infty ,0\leq \sum_{l=0}^{+\infty }k_{4}\left(-l\right)<+\infty . \end{array}$$

## Existence of positive periodic solutions

First we given two notations:

$$\begin{array}{c} I_{\alpha }≔\left\{0,1,2,\cdots ,\alpha -1\right\},\;\bar{\ell }≔\frac{1}{\alpha }\sum_{k=0}^{\alpha -1}\ell \left(k\right), \end{array}$$

where *ℓ*(*k*) is a *α*–periodic sequence of real numbers defined for *k* ∈ *Z*. Let *X*, *Y* be normed vector spaces, *L*: Dom*L* ⊂ *X* → *Y* be a linear mapping, *N*: *X* → *Y* be a continuous mapping. The mapping *L* will be called a Fredholm mapping of index zero if dimKer*L* = codimIm*L* < +∞ and Im*L* be closed in *Y*. If *L* is a Fredholm mapping of index zero and there exist continuous projectors *P*: *X* → *X* and *Q*: *Y* → *Y* such that Im*P* = Ker*L*, Im*L* = Ker*Q* = Im(*I* − *Q*), it follows that *L*∣Dom*L* ∩ Ker*P*: (*I* − *P*)*X* → Im*L* is invertible. We denote the inverse of this map by *K*_*P*_. If Ω is an open bounded subset of *X*, the mapping *N* will be called *L*–compact on $\bar{\Omega }$ if $QN(\bar{\Omega })$ is bounded and $K_{P}\left(I-Q\right)N:\bar{\Omega }\to X$ is compact. Since Im*Q* is isomorphic to Ker*L*, there exists a isomorphism *J*: Im*Q* → Ker*L*.

**Lemma 1** [44] *Let L be a Fredholm mapping of index zero and let N be L*–*compact on* $\bar{\Omega }.$ *If*

*(a)* ∀ *ρ* ∈ (0, 1), *every solution y of Ly* = *ρNy is such that y* ∉ *∂*Ω;

*(b) QNy* ≠ 0, ∀ *x* ∈ *KerL* ⋂ *∂*Ω, *and* deg{*JQN*, Ω ⋂ *KerL*, 0} ≠ 0, *then the equation Ly* = *Ny has at least one solution lying in* $DomL\cap \bar{\Omega }.$

**Lemma 2** [40] *Let h*: *Z* → *R be α periodic, i.e., h*(*k* + *α*) = *h*(*k*), *then* ∀ ς_1_, ς_2_ ∈ *I*_*α*_ *and* ∀ *k* ∈ *Z*, *one has*

$$\begin{array}{c} h\left(k\right)\leq h(\varsigma_{1})+\sum_{s=0}^{\alpha -1}\left|h\left(s+1\right)-g\left(s\right)\right|,\\ h\left(k\right)\geq h(\varsigma_{2})-\sum_{s=0}^{\alpha -1}\left|h\left(s+1\right)-g\left(s\right)\right|. \end{array}$$

**Lemma 3** $\left({\hat{x}}_{1}\left(k\right),{\hat{x}}_{2}\left(k\right)\right)$ *is an α periodic solution of* (4) *with strictly positive components if and only if* $\left(\ln\left\{{\hat{x}}_{1}\left(k\right)\right\},\ln\left\{{\hat{x}}_{2}\left(k\right)\right\}\}\right)$ *is an α periodic solution of*

$$\begin{array}{c} \left\{ \begin{array}{c} x_{1}\left(k+1\right)-x_{1}\left(k\right)=r_{1}\left(k\right)-\sum_{l=0}^{+\infty }k_{1}\left(-l\right)\exp\left(x_{1}\left(k-l\right)\right)\\ \;\;\;\;\;\;\;\;\;\;\;\;\;\;\;\;\;\;\;\;\;\;\;\;\;\;\;\;\;\;\;-\frac{1}{1+m\exp(x_{1}\left(k\right))}\sum_{l=0}^{+\infty }k_{2}\left(-l\right)\exp\left(x_{2}\left(k-l\right)\right),\\ x_{2}\left(k+1\right)-x_{2}\left(k\right)=-r_{2}\left(k\right)-\sum_{l=0}^{+\infty }k_{3}\left(-l\right)\exp\left(x_{2}\left(k-l\right)\right)\\ \;\;\;\;\;\;\;\;\;\;\;\;\;\;\;\;\;\;\;\;\;\;\;\;\;\;\;\;\;\;\;+\sum_{l=0}^{+\infty }\frac{k_{4}\left(-l\right)\exp\left(x_{1}\left(k-l\right)\right)}{1+m\exp(x_{1}\left(k\right))}. \end{array}\right. \end{array}$$ (5)

**Proof** If $\left({\hat{x}}_{1}\left(k\right),{\hat{x}}_{2}\left(k\right)\right)$ is an *α* periodic solution of (4) with strictly positive components, then

$$\begin{array}{c} \left\{ \begin{array}{c} {\hat{x}}_{1}\left(k+1\right)={\hat{x}}_{1}\left(k\right)\exp\{[r_{1}\left(k\right)-\sum_{l=0}^{+\infty }k_{1}\left(-l\right){\hat{x}}_{1}\left(k-l\right)\\ \;\;\;\;\;\;\;\;\;\;\;\;\;\;\;\;-\frac{1}{1+m{\hat{x}}_{1}\left(k\right)}\sum_{l=0}^{+\infty }k_{2}\left(-l\right){\hat{x}}_{2}\left(k-l\right)]\},\\ {\hat{x}}_{2}\left(k+1\right)={\hat{x}}_{2}\left(k\right)\exp\{[-r_{2}\left(k\right)-\sum_{l=0}^{+\infty }k_{3}\left(-l\right){\hat{x}}_{2}\left(k-l\right)\\ \;\;\;\;\;\;\;\;\;\;\;\;\;\;\;\;+\sum_{l=0}^{+\infty }\frac{k_{4}\left(-l\right){\hat{x}}_{1}\left(k-l\right)}{1+m{\hat{x}}_{1}\left(k\right)}]\}. \end{array}\right. \end{array}$$

Hence

$$\begin{array}{c} \left\{ \begin{array}{c} \ln{\hat{x}}_{1}\left(k+1\right)=\ln\{{\hat{x}}_{1}\left(k\right)\exp\{[r_{1}\left(k\right)-\sum_{l=0}^{+\infty }k_{1}\left(-l\right){\hat{x}}_{1}\left(k-l\right)\\ \;\;\;\;\;\;\;\;\;\;\;\;\;\;\;\;-\frac{1}{1+m{\hat{x}}_{1}\left(k\right)}\sum_{l=0}^{+\infty }k_{2}\left(-l\right){\hat{x}}_{2}\left(k-l\right)]\}\},\\ \text{ln}\;{\hat{x}}_{2}\left(k+1\right)=\text{ln}\;\{{\hat{x}}_{2}\left(k\right)\exp\{[-r_{2}\left(k\right)-\sum_{l=0}^{+\infty }k_{3}\left(-l\right){\hat{x}}_{2}\left(k-l\right)\\ \;\;\;\;\;\;\;\;\;\;\;\;\;\;\;\;+\sum_{l=0}^{+\infty }\frac{k_{4}\left(-l\right){\hat{x}}_{1}\left(k-l\right)}{1+m{\hat{x}}_{1}\left(k\right)}]\}\}, \end{array}\right. \end{array}$$

which leads to (5). If $\left(\ln\left\{{\hat{x}}_{1}\left(k\right)\right\},\ln\left\{{\hat{x}}_{2}\left(k\right)\right\}\}\right)$ is an *α* periodic solution of (5), then

$$\begin{array}{c} \left\{ \begin{array}{c} \ln\left\{{\hat{x}}_{1}\left(k+1\right)\right\}-\ln\left\{{\hat{x}}_{1}\left(k\right)\right\}=r_{1}\left(k\right)-\sum_{l=0}^{+\infty }k_{1}\left(-l\right)\exp\left(\ln\left\{{\hat{x}}_{1}\left(k-l\right)\right\}\right)\\ \;\;\;\;\;\;\;\;\;\;\;\;\;\;\;\;\;\;\;\;\;\;\;\;\;\;\;\;\;-\frac{1}{1+m\exp(\ln\left\{{\hat{x}}_{1}\left(k\right)\right\})}\sum_{l=0}^{+\infty }k_{2}\left(-l\right)\exp\left(\ln\left\{{\hat{x}}_{2}\left(k-l\right)\right\}\right),\\ \ln\left\{{\hat{x}}_{2}\left(k+1\right)\right\}-\ln\left\{{\hat{x}}_{2}\left(k\right)\right\}=-r_{2}\left(k\right)-\sum_{l=0}^{+\infty }k_{3}\left(-l\right)\exp\left(\ln\left\{{\hat{x}}_{2}\left(k-l\right)\right\}\right)\\ \;\;\;\;\;\;\;\;\;\;\;\;\;\;\;\;\;\;\;\;\;\;\;\;\;\;\;\;\;+\sum_{l=0}^{+\infty }\frac{k_{4}\left(-l\right)\exp\left(\ln\left\{{\hat{x}}_{1}\left(k-l\right)\right\}\right)}{1+m\exp(\ln\left\{{\hat{x}}_{1}\left(k\right)\right\})}, \end{array}\right. \end{array}$$

which leads to (4).

Define

$$\begin{array}{c} l_{2}=\left\{v=\left\{v\left(k\right)\right\}:v\left(k\right)\in R^{2},k\in Z\right\}. \end{array}$$

Define |*ζ*| = max{|*ζ*_1_|, |*ζ*_2_|}, where *ζ* = (*ζ*_1_, *ζ*_2_)^*T*^ ∈ *R*^2^. Let *l*^*α*^ ⊂ *l*_2_ denote the subspace of all *α* periodic sequences equipped with the norm $∥v∥=\max_{k\in I_{\omega }}\left|v\left(k\right)\right|,$ ∀ *v* = {*v*(*k*):*k* ∈ *Z*} ∈ *l*^*ω*^. Then *l*_*ω*_ is a finite-dimensional Banach space.

Let

$$\begin{array}{c} l_{0}^{\alpha }=\{v=\left\{v\left(k\right)\right\}\in l^{\alpha }:\sum_{k=0}^{\alpha -1}v\left(k\right)=0\},\;\;\;\;\;\;\;\;\;\;\; \end{array}$$ (6)

$$\begin{array}{c} l_{c}^{\alpha }=\{v=\left\{v\left(k\right)\right\}\in l^{\alpha }:v\left(k\right)=h\in R^{2},k\in Z\}, \end{array}$$ (7)

then it follows that $l_{0}^{\alpha }$ and $l_{c}^{\alpha }$ are both closed linear subspaces of *l*^*α*^ and

$$\begin{array}{c} l^{\alpha }=l_{0}^{\alpha }+l_{c}^{\alpha },\;\;\text{dim}l_{c}^{\alpha }=2. \end{array}$$

**Theorem 1** *Let χ*_9_ *be defined by* (32). *Suppose that* (*H*1), (*H*2) *and* $\left(H3\right)\;{\bar{r}}_{1}>\sum_{l=0}^{+\infty }k_{2}\left(-l\right)\exp\left(\chi_{9}\right)$ *hold, then system* (4) *has at least an α periodic solution with positive components*.

**Proof.** Let *X* = *Y* = *l*^*α*^,

$$\left(Lv\right)\left(k\right)=v\left(k+1\right)-v\left(k\right)=\left[\begin{array}{c} x_{1}\left(k+1\right)-x_{1}\left(k\right)\\ x_{2}\left(k+1\right)-x_{2}\left(k\right) \end{array}\right],$$ (8)

$$\left(Nv\right)\left(k\right)=\left[\begin{array}{c} f_{1}\left(k\right)\\ f_{2}\left(k\right) \end{array}\right],$$ (9)

where *v* ∈ *X*, *k* ∈ *Z* and

$$\begin{array}{c} \left\{ \begin{array}{c} f_{1}\left(k\right)=r_{1}\left(k\right)-\sum_{l=0}^{+\infty }k_{1}\left(-l\right)\exp\left(x_{1}\left(k-l\right)\right)\\ \;\;\;\;\;\;\;\;\;\;\;\;\;\;-\frac{1}{1+m\exp(x_{1}\left(k\right))}\sum_{l=0}^{+\infty }k_{2}\left(-l\right)\exp\left(x_{2}\left(k-l\right)\right),\\ f_{2}\left(k\right)=-r_{2}\left(k\right)-\sum_{l=0}^{+\infty }k_{3}\left(-l\right)\exp\left(x_{2}\left(k-l\right)\right)\\ \;\;\;\;\;\;\;\;\;\;\;\;\;\;+\sum_{l=0}^{+\infty }\frac{k_{4}\left(-l\right)\exp\left(x_{1}\left(k-l\right)\right)}{1+m\exp(x_{1}\left(k\right))}. \end{array}\right. \end{array}$$ (10)

Then *L* is a bounded linear operator and

$$\begin{array}{c} \text{Ker}L=l_{c}^{\alpha },\;\;\text{Im}L=l_{0}^{\alpha } \end{array}$$

and

$$\begin{array}{c} \text{dimKer}L=2=\text{codim}\;\text{Im}L, \end{array}$$

then *L* is a Fredholm mapping of index zero. Define

$$\begin{array}{c} Py=\frac{1}{\alpha }\sum_{s=0}^{\alpha -1}y\left(s\right),\;y\in X,\;Qz=\frac{1}{\alpha }\sum_{s=0}^{\omega -1}v\left(s\right),\;v\in Y. \end{array}$$

Then *P* and *Q* are continuous projectors such that

$$\begin{array}{c} \text{Im}P=\text{Ker}L,\;\text{Im}L=\text{Ker}Q=\text{Im}(I-Q). \end{array}$$

In addition, *K*_*P*_: Im*L* → Ker*P* ⋂ Dom*L* exists and

$$\begin{array}{c} K_{P}\left(v\right)=\sum_{s=0}^{\alpha -1}v\left(s\right)-\frac{1}{\alpha }\sum_{s=0}^{\alpha -1}\left(\alpha -s\right)v\left(s\right). \end{array}$$

By the equation *Lv* = *ρNv*, *ρ* ∈ (0, 1), one gets

$$\begin{array}{c} \left\{ \begin{array}{c} x_{1}\left(k+1\right)-x_{1}\left(k\right)=\rho [r_{1}\left(k\right)-\sum_{l=0}^{+\infty }k_{1}\left(-l\right)\exp\left(x_{1}\left(k-l\right)\right)\\ \;\;\;\;\;\;\;\;\;\;\;\;\;\;\;\;\;\;\;\;\;\;\;\;\;\;\;\;\;\;\;-\frac{1}{1+m\exp(x_{1}\left(k\right))}\sum_{l=0}^{+\infty }k_{2}\left(-l\right)\exp\left(x_{2}\left(k-l\right)\right)],\\ x_{2}\left(k+1\right)-x_{2}\left(k\right)=\rho [-r_{2}\left(k\right)-\sum_{l=0}^{+\infty }k_{3}\left(-l\right)\exp\left(x_{2}\left(k-l\right)\right)\\ \;\;\;\;\;\;\;\;\;\;\;\;\;\;\;\;\;\;\;\;\;\;\;\;\;\;\;\;\;\;\;+\sum_{l=0}^{+\infty }\frac{k_{4}\left(-l\right)\exp\left(x_{1}\left(k-l\right)\right)}{1+m\exp(x_{1}\left(k\right))}]. \end{array}\right. \end{array}$$ (11)

Suppose that *v*(*k*) = (*x*_1_(*k*), *x*_2_(*k*))^*T*^ ∈ *X* is an arbitrary solution of system (11) for a certain *ρ* ∈ (0, 1) then one has

$$\begin{array}{ccc} & \; & \sum_{k=0}^{\alpha -1}[\sum_{l=0}^{+\infty }k_{1}\left(-l\right)\exp\left(x_{1}\left(k-l\right)\right)\\ & \; & \;+\frac{1}{1+m\exp(x_{1}\left(k\right))}\sum_{l=0}^{+\infty }k_{2}\left(-l\right)\exp\left(x_{2}\left(k-l\right)\right)]={\bar{r}}_{1}\alpha , \end{array}$$ (12)

$$\begin{array}{ccc} & \; & \sum_{k=0}^{\alpha -1}[\sum_{l=0}^{+\infty }k_{3}\left(-l\right)\exp\left(x_{2}\left(k-l\right)\right)-\sum_{l=0}^{+\infty }\frac{k_{4}\left(-l\right)\exp\left(x_{1}\left(k-l\right)\right)}{1+m\exp(x_{1}\left(k\right))}]={\bar{r}}_{2}\alpha . \end{array}$$ (13)

It follows from (11)–(13) that

$$\begin{array}{c} \sum_{k=0}^{\alpha -1}\left|x_{1}\left(k+1\right)-x_{1}\left(k\right)\right|\leq 2{\bar{r}}_{1}\alpha , \end{array}$$ (14)

$$\begin{array}{c} \sum_{k=0}^{\alpha -1}\left|x_{2}\left(k+1\right)-x_{2}\left(k\right)\right|\leq 2{\bar{r}}_{2}\alpha . \end{array}$$ (15)

If *v* = {*v*(*k*)} ∈ *X*, then ∃ *ξ*_*i*_, *η*_*i*_ ∈ *I*_*α*_ such that

$$\begin{array}{c} x_{i}\left(\xi_{i}\right)=\min_{k\in I_{\alpha }}\left\{x_{i}\left(k\right)\right\},\;x_{i}\left(\eta_{i}\right)=\max_{k\in I_{\alpha }}\left\{x_{i}\left(k\right)\right\}\left(i=1,2\right). \end{array}$$ (16)

By (12) and (13), we have

$$\begin{array}{c} \sum_{l=0}^{+\infty }k_{1}\left(-l\right)\exp\left(x_{1}\left(\xi_{1}\right)\right)\leq \sum_{l=0}^{+\infty }k_{1}\left(-l\right)\exp\left(x_{1}\left(k-l\right)\right)<{\bar{r}}_{1}\alpha , \end{array}$$ (17)

$$\begin{array}{c} \sum_{l=0}^{+\infty }k_{3}\left(-l\right)\exp\left(x_{2}\left(\eta_{2}\right)\right)\geq \sum_{l=0}^{+\infty }k_{3}\left(-l\right)\exp\left(x_{2}\left(k-l\right)\right)>{\bar{r}}_{2}\alpha . \end{array}$$ (18)

Thus

$$\begin{array}{c} x_{1}\left(\xi_{1}\right)<\ln[\frac{{\bar{r}}_{1}}{\sum_{l=0}^{+\infty }k_{1}\left(-l\right)}]≔\theta_{1}, \end{array}$$ (19)

$$\begin{array}{c} x_{2}\left(\eta_{2}\right)>\ln[\frac{{\bar{r}}_{2}}{\sum_{l=0}^{+\infty }k_{3}\left(-l\right)}]≔\delta_{2}. \end{array}$$ (20)

In the sequel, we consider two cases.

**(a)** If *x*_1_(*η*_1_) ≥ *x*_2_(*η*_2_), then it follows from (12) that

$$\begin{array}{c} {}[\sum_{l=0}^{+\infty }k_{1}\left(-l\right)+\sum_{l=0}^{+\infty }k_{2}\left(-l\right)]\exp\left(x_{1}\left(\eta_{1}\right)\right)\alpha \geq {\bar{r}}_{1}\alpha \end{array}$$

which leads to

$$\begin{array}{c} x_{1}\left(\eta_{1}\right)>\ln[\frac{{\bar{r}}_{1}}{\sum_{l=0}^{+\infty }k_{1}\left(-l\right)+\sum_{l=0}^{+\infty }k_{2}\left(-l\right)}]≔\theta_{2}, \end{array}$$ (21)

It follows from (19),(21) and Lemma 2 that

$$\begin{array}{ccc} x_{1}\left(k\right) & \leq & x_{1}\left(\xi_{1}\right)+\sum_{s=0}^{\alpha -1}\left|x_{1}\left(s+1\right)-x_{1}\left(s\right)\right|\\ & \leq & \theta_{1}+2{\bar{r}}_{1}\alpha ≔\chi_{1}, \end{array}$$ (22)

$$\begin{array}{ccc} x_{1}\left(k\right) & \geq & x_{1}\left(\eta_{1}\right)-\sum_{s=0}^{\alpha -1}\left|x_{1}\left(s+1\right)-x_{1}\left(s\right)\right|\\ & \geq & \theta_{2}-2{\bar{r}}_{1}\alpha ≔\chi_{2}. \end{array}$$ (23)

By (22) and (23), we derive

$$\begin{array}{c} \max_{k\in I_{\alpha }}\left\{x_{1}\left(k\right)\right\}\leq \max\{|\chi_{1}\left|,\right|\chi_{2}\left|\right\}≔\chi_{3}. \end{array}$$ (24)

From (13) and (24), we obtain

$$\begin{array}{c} \sum_{l=0}^{+\infty }k_{3}\left(-l\right)\exp\left(x_{2}\left(\xi_{2}\right)\right)\alpha -\sum_{l=0}^{+\infty }k_{4}\left(-l\right)\exp\left(\chi_{3}\right)\alpha \leq {\bar{r}}_{2}\alpha . \end{array}$$

Then

$$\begin{array}{c} x_{2}\left(\xi_{2}\right)\leq \ln[\frac{{\bar{r}}_{2}+\sum_{l=0}^{+\infty }k_{4}\left(-l\right)\exp\left(\chi_{3}\right)}{\sum_{l=0}^{+\infty }k_{3}\left(-l\right)}]≔\delta_{1}. \end{array}$$ (25)

Thus by (20), (25) and Lemma 3.2, we get

$$\begin{array}{ccc} x_{2}\left(k\right) & \leq & x_{2}\left(\xi_{2}\right)+\sum_{s=0}^{\alpha -1}\left|x_{2}\left(s+1\right)-x_{2}\left(s\right)\right|\\ & \leq & \delta_{1}+2{\bar{r}}_{2}\alpha ≔\chi_{4}, \end{array}$$ (26)

$$\begin{array}{ccc} x_{2}\left(k\right) & \geq & x_{2}\left(\eta_{2}\right)-\sum_{s=0}^{\alpha -1}\left|x_{2}\left(s+1\right)-x_{2}\left(s\right)\right|\\ & \geq & \delta_{2}-2{\bar{r}}_{2}\alpha ≔\chi_{5}. \end{array}$$ (27)

It follows from (26) and (27) that

$$\begin{array}{c} \max_{k\in I_{\alpha }}\left\{x_{2}\left(k\right)\right\}\leq \max\{|\chi_{4}\left|,\right|\chi_{5}\left|\right\}≔\chi_{6}. \end{array}$$ (28)

**(b)** If *x*_1_(*η*_1_) < *x*_2_(*η*_2_), then it follows from (13) that

$$\begin{array}{c} \sum_{l=0}^{+\infty }k_{3}\left(-l\right)\exp\left(x_{2}\left(\xi_{2}\right)\right)\alpha -\frac{1}{m}\sum_{l=0}^{+\infty }k_{4}\left(-l\right)\alpha \leq {\bar{r}}_{2}\alpha \end{array}$$

which leads to

$$\begin{array}{c} x_{2}\left(\xi_{2}\right)<\ln[\frac{{\bar{r}}_{2}+\frac{1}{m}\sum_{l=0}^{+\infty }k_{4}\left(-l\right)}{\sum_{l=0}^{+\infty }k_{3}\left(-l\right)}]≔{\bar{\delta }}_{1}, \end{array}$$ (29)

It follows from (20),(29) and Lemma 3.2 that

$$\begin{array}{ccc} x_{2}\left(k\right) & \leq & x_{2}\left(\xi_{2}\right)+\sum_{s=0}^{\alpha -1}\left|x_{2}\left(s+1\right)-x_{2}\left(s\right)\right|\\ & \leq & {\bar{\delta }}_{1}+2{\bar{r}}_{2}\alpha ≔\chi_{7}, \end{array}$$ (30)

$$\begin{array}{ccc} x_{2}\left(k\right) & \geq & x_{2}\left(\eta_{2}\right)-\sum_{s=0}^{\alpha -1}\left|x_{2}\left(s+1\right)-x_{2}\left(s\right)\right|\\ & \geq & \delta_{2}-2{\bar{r}}_{2}\alpha ≔\chi_{8}. \end{array}$$ (31)

By (30) and (31), we derive

$$\begin{array}{c} \max_{k\in I_{\alpha }}\left\{x_{2}\left(k\right)\right\}\leq \max\{|\chi_{7}\left|,\right|\chi_{8}\left|\right\}≔\chi_{9}. \end{array}$$ (32)

From (12), we get

$$\begin{array}{c} {}[\sum_{l=0}^{+\infty }k_{1}\left(-l\right)\exp\left(x_{1}\left(\eta_{1}\right)\right)\alpha +\sum_{l=0}^{+\infty }k_{2}\left(-l\right)\exp\left(\chi_{9}\right)\alpha ]\geq {\bar{r}}_{1}\alpha . \end{array}$$

Then

$$\begin{array}{c} x_{1}\left(\eta_{1}\right)\geq \ln[\frac{{\bar{r}}_{1}-\sum_{l=0}^{+\infty }k_{2}\left(-l\right)\exp\left(\chi_{9}\right)}{\sum_{l=0}^{+\infty }k_{1}\left(-l\right)}]≔{\bar{\theta }}_{2}. \end{array}$$ (33)

Thus by (19), (33) and Lemma 3.2, we get

$$\begin{array}{ccc} x_{1}\left(k\right) & \leq & x_{1}\left(\xi_{1}\right)+\sum_{s=0}^{\alpha -1}\left|x_{1}\left(s+1\right)-x_{1}\left(s\right)\right|\\ & \leq & \delta_{1}+2{\bar{r}}_{2}\alpha ≔\chi_{10}, \end{array}$$ (34)

$$\begin{array}{ccc} x_{1}\left(k\right) & \geq & x_{1}\left(\eta_{1}\right)-\sum_{s=0}^{\alpha -1}\left|x_{1}\left(s+1\right)-x_{1}\left(s\right)\right|\\ & \geq & {\bar{\theta }}_{2}-2{\bar{r}}_{2}\alpha ≔\chi_{11}. \end{array}$$ (35)

It follows from (34) and (35) that

$$\begin{array}{c} \max_{k\in I_{\alpha }}\left\{x_{1}\left(k\right)\right\}\leq \max\{|\chi_{10}\left|,\right|\chi_{11}\left|\right\}≔\chi_{12}. \end{array}$$ (36)

Then *χ*_*i*_(*i* = 1, 2, ⋯, 11) has no relation with *ρ* ∈ (0, 1). Let *M* = max{*χ*_3_, *χ*_6_, *χ*_9_, *χ*_12_} + *M*_0_, where *M*_0_ > 0 which satisfies $\max\{|\ln\left\{x_{1}^{*}\right\}|,|\ln\left\{x_{2}^{*}\right\}\left|\right\}<M_{0}$, where ${\left(x_{1}^{*},x_{2}^{*}\right)}^{T}$ is the unique positive solution of (5). Thus any solution *v* = {*v*(*k*)} = {(*x*_1_(*k*), *x*_2_(*k*))^*T*^} of (11) in *X* satisfies ‖*v*‖ < *M*, *k* ∈ *Z*.

Let Ω ≔ {*v* = {*v*(*k*)} ∈ *X*: ‖*v*‖ < *M*}, then Ω is an open, bounded set in *X* and (a) of Lemma 1 is satisfied. When *v* ∈ ∂Ω ∩ Ker*L*, *v* = {(*x*_1_, *x*_2_)^*T*^} with ‖*v*‖ = max{|*x*_1_|, |*x*_2_|} = *M*. Then

$$\begin{array}{c} QNz=[\begin{array}{c} \Lambda_{1}\\ \Lambda_{2} \end{array}]\neq 0, \end{array}$$

where

$$\begin{array}{ccc} & & \Lambda_{1}={\bar{r}}_{1}-\sum_{l=0}^{+\infty }k_{1}\left(-l\right)\exp\left(x_{1}\right)-\sum_{k=0}^{\alpha -1}\frac{1}{1+m\exp(x_{1})}\sum_{l=0}^{+\infty }k_{2}\left(-l\right)\exp\left(x_{2}\right),\\ & & \Lambda_{2}=-{\bar{r}}_{2}-\sum_{l=0}^{+\infty }k_{3}\left(-l\right)\exp\left(x_{2}\right)+\sum_{k=0}^{\alpha -1}\sum_{l=0}^{+\infty }\frac{k_{4}\left(-l\right)\exp\left(x_{1}\right)}{1+m\exp(x_{1})}. \end{array}$$

Let *ϕ*(*x*_1_, *x*_2_, *μ*) = *μQNv* + (1 − *μ*)*Gv*, *μ* ∈ [0, 1], where

$$\begin{array}{c} Gv=(\begin{array}{c} {\bar{r}}_{1}-\sum_{l=0}^{+\infty }k_{1}\left(-l\right)\exp\left(x_{1}\right)\\ -{\bar{r}}_{2}-\sum_{l=0}^{+\infty }k_{3}\left(-l\right)\exp\left(x_{2}\right) \end{array}). \end{array}$$

Letting *J* be the identity mapping, we have

$$\begin{array}{ccc} & & \deg[JQN{\left(x_{1},x_{2}\right)}^{T};\Omega \cap \text{ker}L;0]\\ & & =\deg[QN{\left(x_{1},x_{2}\right)}^{T};\Omega \cap \text{ker}L;0]\\ & & =\deg[\phi (x_{1},x_{2},1);\Omega \cap \text{ker}L;0]\\ & & =\deg[\phi (x_{1},x_{2},0);\Omega \cap \text{ker}L;0]\\ & & =\text{sign}\{\det[\begin{array}{cc} \sum_{l=0}^{+\infty }k_{1}\left(-l\right)\exp\left(x_{1}^{*}\right) & 0\\ 0 & -\sum_{l=0}^{+\infty }k_{3}\left(-l\right)\exp\left(x_{2}^{*}\right) \end{array}]\}\\ & & =\text{sign}[-\sum_{l=0}^{+\infty }k_{1}\left(-l\right)\sum_{l=0}^{+\infty }k_{3}\left(-l\right)\exp\left(x_{2}^{*}\right)\exp\left(x_{1}^{*}+x_{2}^{*}\right)]=-1\neq 0. \end{array}$$

It follows that *Lv* = *Nv* has at least one solution in $\text{Dom}L\cap \bar{\Omega }$, i.e., (5) has at least one *α* periodic solution in $\text{Dom}L\cap \bar{\Omega }$, say $v^{*}=\left\{v^{*}\left(k\right)\right\}=\left\{{\left(x_{1}^{*}\left(k\right),x_{2}^{*}\left(k\right)\right)}^{T}\right\}$. Let ${\bar{x}}_{1}^{*}\left(k\right)=\exp\left\{x_{1}^{*}\left(k\right)\right\},{\bar{x}}_{2}^{*}\left(k\right)=\exp\left\{x_{2}^{*}\left(k\right)\right\}\}$ then by Lemma 3 we know that ${\bar{v}}^{*}=\left\{{\bar{x}}^{*}\left(k\right)\right\}={\left\{{\bar{x}}_{1}^{*}\left(k\right),{\bar{x}}_{2}^{*}\left(k\right)\right)}^{T}\}$ is a *α* positive periodic solution of system (4). The proof is complete.

## Global asymptotic stability

Let the delays be zero, then (4) becomes

$$\begin{array}{c} \left\{ \begin{array}{c} x_{1}\left(k+1\right)=x_{1}\left(k\right)\exp\{[r_{1}\left(k\right)-k_{1}x_{1}\left(k\right)-\frac{k_{2}x_{2}\left(k\right)}{1+mx_{1}\left(k\right)}]\},\\ x_{2}\left(k+1\right)=x_{2}\left(k\right)\exp\{[-r_{2}\left(k\right)-k_{3}x_{2}\left(k\right)+\frac{k_{4}x_{1}\left(k\right)}{1+mx_{1}\left(k\right)}]\}. \end{array}\right. \end{array}$$ (37)

**Theorem 2** *Assume that* (H1) *and* (H2) *are satisfied and furthermore suppose that there exist positive constants ν*, *σ*_1_ *and σ*_2_ *such that*

$$\begin{array}{c} \left\{ \begin{array}{c} \sigma_{1}[k_{1}+\frac{k_{2}m}{{\left(1+mx_{1}^{*}\right)}^{2}}]-\sigma_{2}[\frac{1}{{\left(1+mx_{1}^{*}\right)}^{2}}]>\nu ,\\ \sigma_{2}k_{3}-\sigma_{1}[\frac{mx_{1}^{*}\left(k\right)x_{2}^{*}\left(k\right)}{{\left(1+mx_{1}^{*}\right)}^{2}}]>\nu . \end{array}\right. \end{array}$$ (38)

*Then the positive ω*-*periodic solution of system* (37) *is globally asymptotically stable*.

**Proof** In view of Theorem 1, there exists a positive periodic solution $\left\{x_{1}^{*}\left(k\right),x_{2}^{*}\left(k\right)\right\}$ of system (37). Make the change of variable

$$\begin{array}{c} u_{i}\left(k\right)=x_{i}\left(k\right)-x_{i}^{*}\left(k\right)\left(i=1,2\right). \end{array}$$ (39)

It follows from (37) that

$$\begin{array}{ccc} u_{1}\left(k+1\right) & = & x_{1}\left(k+1\right)-x_{1}^{*}\left(k+1\right)\\ & = & x_{1}\left(k\right)\exp\{[r_{1}\left(k\right)-k_{1}x_{1}\left(k\right)-\frac{k_{2}x_{2}\left(k\right)}{1+mx_{1}\left(k\right)}]\}\\ & \; & -x_{1}^{*}\left(k\right)\exp\{[r_{1}\left(k\right)-\sum_{l=0}^{+\infty }k_{1}\left(-l\right)x_{1}^{*}\left(k\right)-\frac{k_{2}x_{2}^{*}\left(k\right)}{1+mx_{1}^{*}\left(k\right)}]\}\\ & = & \{x\left(k\right)\exp[-(k_{1}+\frac{k_{2}mx_{1}^{*}\left(k\right)}{{\left(1+mx_{1}^{*}\right)}^{2}})u_{1}\left(k\right)\\ & \; & -(\frac{k_{2}}{1+mx_{1}^{*}\left(k\right)})u_{2}\left(k\right)]-x^{*}\left(k\right)\}\frac{x^{*}\left(k+1\right)}{x^{*}\left(k\right)}\\ & = & \{[1-(k_{1}x_{1}^{*}\left(k\right)+\frac{k_{2}mx_{1}^{*}\left(k\right)}{{\left(1+mx_{1}^{*}\right)}^{2}})]\frac{u_{1}\left(k\right)}{x^{*}\left(k\right)}\\ & \; & -(\frac{mx_{1}^{*}\left(k\right)x_{2}^{*}\left(k\right)}{{\left(1+mx_{1}^{*}\right)}^{2}})u_{2}\left(k\right)+\gamma_{1}\}x^{*}\left(k+1\right), \end{array}$$ (40)

$$\begin{array}{ccc} u_{2}\left(k+1\right) & = & x_{2}\left(k+1\right)-x_{2}^{*}\left(k+1\right)\\ & = & x_{2}\left(k\right)\exp\{[-r_{2}\left(k\right)-k_{3}x_{2}\left(k\right)+\frac{k_{4}x_{1}\left(k\right)}{1+mx_{1}\left(k\right)}]\}\\ & \; & -x_{2}^{*}\left(k\right)\exp\{[-r_{2}\left(k\right)-k_{3}x_{2}^{*}\left(k\right)+\frac{k_{4}x_{1}^{*}\left(k\right)}{1+mx_{1}\left(k\right)}]\}\\ & = & \left\{x_{2}\left(k\right)\exp[-k_{3}u_{2}\left(k\right)-(\frac{k_{4}x_{1}\left(k\right)}{1+mx_{1}\left(k\right)}-\frac{k_{4}x_{1}^{*}\left(k\right)}{1+mx_{1}\left(k\right)})]-x_{2}^{*}\left(k\right)\right\}\\ & \; & \times \frac{y^{*}\left(k+1\right)}{x_{2}^{*}\left(k\right)}\\ & = & \{[1-k_{3}x_{2}^{*}\left(k\right)]\frac{u_{2}\left(k\right)}{x_{2}^{*}\left(k\right)}-\frac{1}{{\left(1+mx_{1}^{*}\right)}^{2}}u_{1}\left(k\right)+\gamma_{2}\}x_{2}^{*}\left(k+1\right). \end{array}$$ (41)

where $\frac{\left|\right|\gamma_{i}\left|\right|}{\left||u|\right|}\left(i=1,2\right)$ converges to zero as ||*u*|| → 0.

Define a function *V* by

$$\begin{array}{c} V\left(N\left(k\right)\right)=\sigma_{1}|\frac{u_{1}\left(k\right)}{x_{1}^{*}\left(k\right)}|+\sigma_{2}|\frac{u_{2}\left(k\right)}{x_{2}^{*}\left(k\right)}|, \end{array}$$ (42)

where *σ*_1_ > 0 and *σ*_2_ > 0 are given by (44) and (45) respectively. Calculating the difference of *V* along the solution of system (40) and (41), we have

$$\begin{array}{ccc} \Delta V & = & \sigma_{1}(|\frac{u_{1}\left(k+1\right)}{x_{1}^{*}\left(k+1\right)}-\frac{u_{1}\left(k\right)}{x_{1}^{*}\left(k\right)}|)+\sigma_{2}(|\frac{u_{2}\left(k+1\right)}{x_{2}^{*}\left(k+1\right)}-\frac{u_{2}\left(k\right)}{x_{2}^{*}\left(k\right)}|)\\ & \leq & -\sigma_{1}[k_{1}+\frac{k_{2}m}{{\left(1+mx_{1}^{*}\right)}^{2}}]|u_{1}\left(k\right)|+\sigma_{1}[\frac{mx_{1}^{*}\left(k\right)x_{2}^{*}\left(k\right)}{{\left(1+mx_{1}^{*}\right)}^{2}}]\left|u_{2}\left(k\right)\right|\\ & \; & -\sigma_{2}k_{3}|u_{2}\left(k\right)|+\sigma_{2}[\frac{1}{{\left(1+mx_{1}^{*}\right)}^{2}}]\left|u_{1}\left(k\right)\right|\\ & \leq & -\Pi_{1}|u_{1}\left(k\right)|-\Pi_{2}\left|u_{2}\left(k\right)\right|, \end{array}$$ (43)

where

$$\Pi_{1}=\sigma_{1}\left[k_{1}+\frac{k_{2}m}{{\left(1+mx_{1}^{*}\right)}^{2}}\right]-\sigma_{2}\left[\frac{1}{{\left(1+mx_{1}^{*}\right)}^{2}}\right],$$ (44)

$$\Pi_{2}=\sigma_{2}k_{3}-\sigma_{1}\left[\frac{mx_{1}^{*}\left(k\right)x_{2}^{*}\left(k\right)}{{\left(1+mx_{1}^{*}\right)}^{2}}\right].$$ (45)

It follows from the condition (38) that ∃ *ϵ* > 0 such that, if *k* is sufficiently large and ||*u*|| < *ϵ*, then

$$\begin{array}{c} \Delta V\leq -\frac{\nu }{2}\left\{\right|u_{1}\left(k\right)|+|u_{2}\left(k\right)\left|\right\}<-\frac{\nu \epsilon }{2}. \end{array}$$ (46)

In view of Freedman [45], we can see that the trivial solutions of (40) and (41) is uniformly asymptotically stable and so is the solution {(x*(*k*), y*(*k*))^*T*^} of (37). The proof is complete.

**Remark 1** *In* [34], *Ye et al. investigated the periodic solution of a continuous predator-prey system with Holling type II functional response and infinite delays by applying continuation theorem in coincidence degree theory and some priori estimates on solutions, moreover, this paper does not involve the global asymptotic stability. In this paper, we study the existence of periodic solution of discrete predator-prey model with distributed delays by applying continuation theorem in coincidence degree theory and analyze the global asymptotic stability of periodic solution by Lyapunov function. Form this viewpoint, the results of this article supplement the previous studies of Ye et al*. [18].

## Numerical example

**Fig 1:**
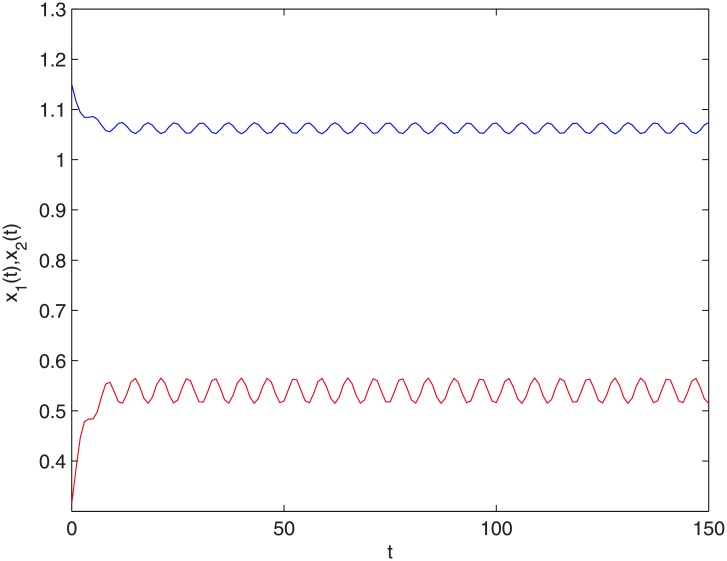
The time histories of *t*-*x*_1_,*t*-*x*_2_. The blue line stands for *x*_1_(*t*) and the red line stands for *x*_2_(*t*).

**Fig 2:**
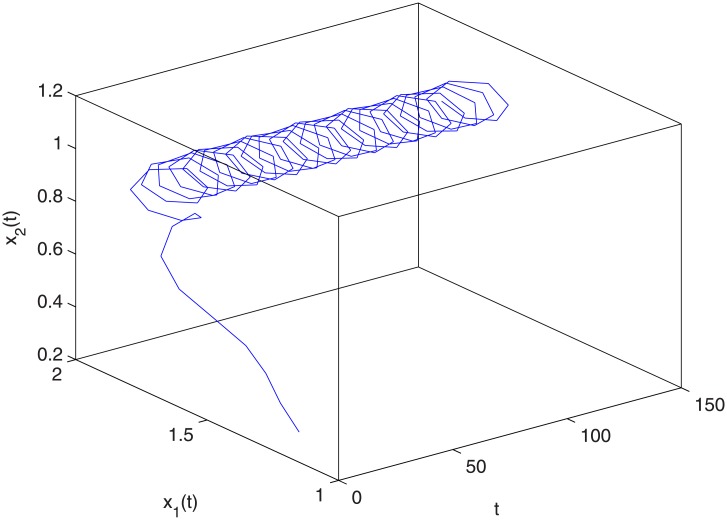
The relational graph of *t*, *x*_1_ and *x*_2_.

**Example 1** Consider the model as follows:

$$\begin{array}{c} \left\{ \begin{array}{c} x_{1}\left(k+1\right)=x_{1}\left(k\right)\exp\{[r_{1}\left(k\right)-\sum_{l=0}^{+\infty }k_{1}\left(-l\right)x_{1}\left(k-l\right)\\ \;\;\;\;\;\;\;\;\;\;\;\;\;\;\;\;-\frac{1}{1+mx_{1}\left(k\right)}\sum_{l=0}^{+\infty }k_{2}\left(-l\right)x_{2}\left(k-l\right)]\},\\ x_{2}\left(k+1\right)=x_{2}\left(k\right)\exp\{[-r_{2}\left(k\right)-\sum_{l=0}^{+\infty }k_{3}\left(-l\right)x_{2}\left(k-l\right)\\ \;\;\;\;\;\;\;\;\;\;\;\;\;\;\;\;+\sum_{l=0}^{+\infty }\frac{k_{4}\left(-l\right)x_{1}\left(k-l\right)}{1+mx_{1}\left(k\right)}]\}, \end{array}\right. \end{array}$$ (47)

where *r*_1_(*k*) = 0.6 + sin *kπ*, *r*_2_(*k*) = 0.45 + sin *kπ*, *m* = 5, *k*_*i*_(*s*) = *e*^*s*^(*i* = 1, 2, 3, 4). So ${\bar{r}}_{1}=0.3,{\bar{r}}_{2}=0.225,\sum_{l=0}^{+\infty }k_{2}\left(-l\right)\exp\left(\chi_{9}\right)\approx 0.2437.$ Thus the conditions (H1)-(H3) of Theorem 3.1 hold true. Therefore, system (47) has at least a positive two-periodic solution (see Figs 1 and 2). Fig 1 shows the changing situation of prey density with the increase of time *t*; Fig 2 shows the changing situation of predator density with the increase of time *t*; From Figs 1 and 2, we can see that the prey density and the predator density will keep periodic oscillation with the increase of time *t*.

**Fig 3:**
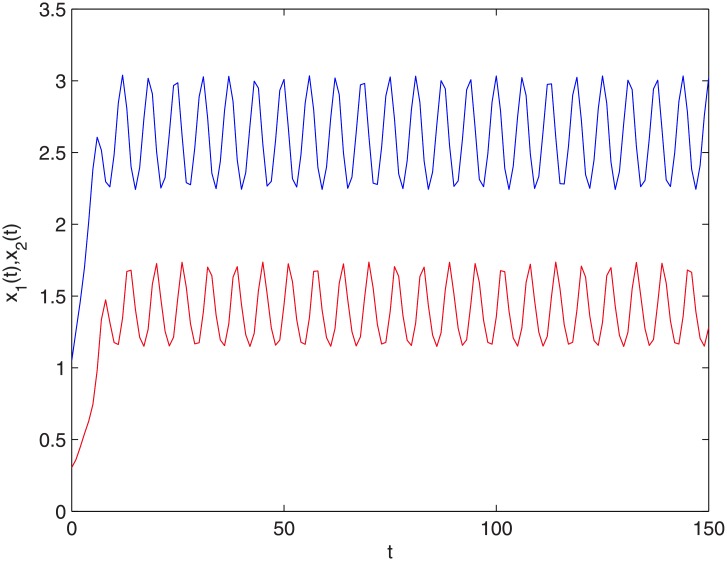
The time histories of *t*-*x*_1_,*t*-*x*_2_. The blue line stands for *x*_1_(*t*) and the red line stands for *x*_2_(*t*).

**Fig 4:**
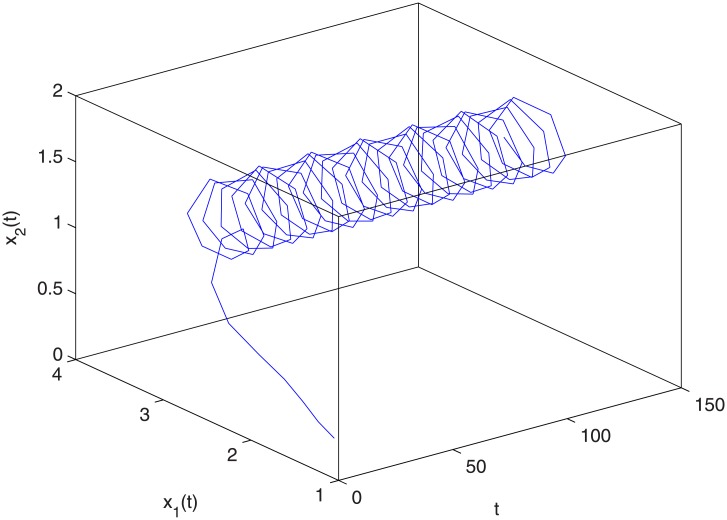
The relational graph of *t*, *x*_1_ and *x*_2_.

**Example 2** Consider the model as follows:

$$\begin{array}{c} \left\{ \begin{array}{c} x_{1}\left(k+1\right)=x_{1}\left(k\right)\exp\{[r_{1}\left(k\right)-k_{1}x_{1}\left(k\right)-\frac{k_{2}x_{2}\left(k\right)}{1+mx_{1}\left(k\right)}]\},\\ x_{2}\left(k+1\right)=x_{2}\left(k\right)\exp\{[-r_{2}\left(k\right)-k_{3}x_{2}\left(k\right)+\frac{k_{4}x_{1}\left(k\right)}{1+mx_{1}\left(k\right)}]\}. \end{array}\right. \end{array}$$ (48)

where *r*_1_(*k*) = 0.2 + cos *kπ*, *r*_2_(*k*) = 0.1 + cos *kπ*, *m* = 2, *k*_1_ = 0.2, *k*_2_ = 0.12, *k*_3_ = 0.24, *k*_4_ = 0.35,. So ${\bar{r}}_{1}=0.1,{\bar{r}}_{2}=0.05.$ Let *σ*_1_ = 0.18, *σ*_2_ = 0.23, *ν* = 0.04. Thus the conditions (H1)-(H2) and (38) of Theorem 4.1 are satisfied. Thus the positive two-periodic solution of system (48) is globally asymptotically stable (see Figs 3 and 4). Fig 3 shows the changing situation of prey density with the increase of time *t*; Fig 4 shows the changing situation of predator density with the increase of time *t*; From Figs 3 and 4, we can see that the prey density and the predator density will keep globally asymptotically stable periodic oscillation with the increase of time *t*.

## Conclusions

Based on the previous works and some biological meanings of predators and preys, we propose a new discrete delayed predator-prey system. By using the continuation theorem in coincidence degree theory, we present a set of sufficient conditions to ensure to ensure the existence of positive periodic solution of the discrete delayed predator-prey system. In addition, we also discussed the global asymptotic stability of positive periodic solution for the considered system. The obtained theoretical findings have important significance in biological ecology. Considering the effect of random factor, it is meaningful for us to deal with the dynamics of stochastic predator-prey system. This topic will be our future research direction.

## Supporting information

S1 Fig The time histories of *t*-*x*_1_,*t*-*x*_2_. The blue line stands for *x*_1_(*t*) and the red line stands for *x*_2_(*t*).
(DOC)

S2 Fig The relational graph of *t*, *x*_1_ and *x*_2_. (DOC)

S3 Fig The time histories of *t*-*x*_1_,*t*-*x*_2_. The blue line stands for *x*_1_(*t*) and the red line stands for *x*_2_(*t*).
(DOC)

S4 Fig The relational graph of *t*, *x*_1_ and *x*_2_. (DOC)
