# Supplementary material for: Oscillatory dynamics in a discrete predator-prey model with distributed delays
Source: PLoS One. 2018 Dec 26;13(12):e0208322. doi: 10.1371/journal.pone.0208322 (PMC6306177; doi:10.1371/journal.pone.0208322)
Supplement: S3 Fig — The blue line stands for x1(t) and the red line stands for x2(t). (DOC) [file pone.0208322.s003.doc]

S3 Fig. The time histories of t-x_1 ,t-x_2. The blue line stands for x_1(t) and the red line stands for x_2(t).

x0=1.05

y0=0.305;

n=150;

x=zeros(n+1,1);

y=zeros(n+1,1);

t=zeros(n+1,1);

x(1)=x0;

y(1)=y0;

for i=1:n

t(i)=i-1;

x(i+1)= x(i)*exp(0.3+0.03*sin(i)-(0.04+0.04*sin(i))*x(i)-(0.02+0.03*sin(i))*y(i)-0.02*(x(i)+x(i))-0.02*(y(i)+y(i)));

y(i+1)=y(i)*exp(-0.027-0.003*sin(i)+(0.26+0.05*sin(i))*x(i)-(0.5+0.02*sin(i))*y(i)+0.03*(x(i)+x(i))-0.04*(y(i)+y(i)));

end

t(n+1)=n;

plot(t,y,t,x,t,x,'b',t,y,'r')

xlabel('t'),ylabel('x_1(t),x_2(t) ')
